# Downregulation and Mutation of a Cadherin Gene Associated with Cry1Ac Resistance in the Asian Corn Borer, *Ostrinia furnacalis* (Guenée)

**DOI:** 10.3390/toxins6092676

**Published:** 2014-09-11

**Authors:** Tingting Jin, Xue Chang, Angharad M. R. Gatehouse, Zhenying Wang, Martin G. Edwards, Kanglai He

**Affiliations:** 1 The State Key Laboratory for Biology of Plant Diseases and Insect Pests, Institute of Plant Protection, Chinese Academy of Agricultural Sciences, Beijing 100193, China; E-Mails: jintingting492@126.com (T.T.J.); cx1981@cjaas.com (X.C.); zywang@ippcaas.cn (Z.W.); 2 Newcastle Institute for Research on Sustainability, School of Biology, University of Newcastle, Newcastle upon Tyne NE1 7RU, UK; E-Mails: a.m.r.gatehouse@newcastle.ac.uk (A.M.R.G.); martin.edwards@newcastle.ac.uk (M.G.E.)

**Keywords:** *Ostrinia furnacalis*, *Bacillus thuringiensis*, Cry1Ac toxin, resistance, cadherin, binding receptor

## Abstract

Development of resistance in target pests is a major threat to long-term use of transgenic crops expressing *Bacillus thuringiensis* (*Bt*) Cry toxins. To manage and/or delay the evolution of resistance in target insects through the implementation of effective strategies, it is essential to understand the basis of resistance. One of the most important mechanisms of insect resistance to *Bt* crops is the alteration of the interactions between Cry toxins and their receptors in the midgut. A Cry1Ac-selected strain of Asian corn borer (ACB), *Ostrinia furnacalis*, a key pest of maize in China, evolved three mutant alleles of a cadherin-like protein (OfCAD) (MPR-r1, MPR-r2 and MPR-r3), which mapped within the toxin-binding region (TBR). Each of the three mutant alleles possessed two or three amino acid substitutions in this region, especially Thr^1457^→Ser. In highly resistant larvae (ACB-Ac200), MPR-r2 had a 26-amino acid residue deletion in the TBR, which resulted in reduced binding of Cry1Ac compared to the MPR from the susceptible strain, suggesting that the number of amino acid deletions influences the level of resistance. Furthermore, downregulation of OfCAD gene (*ofcad*) transcription was observed in the Cry1Ac resistant strain, ACB-Ac24, suggesting that Cry1Ac resistance in ACB is associated with the downregulation of the transcript levels of the cadherin-like protein gene. The OfCAD identified from ACB exhibited a high degree of similarity to other members of the cadherin super-family in lepidopteran species.

## 1. Introduction

Insecticidal Cry toxins, which are generated by *Bacillus thuringiensis* as protein crystals, composed of three discrete functional domains, are widely used to control insect pests in agriculture and important disease vectors in public health [1]. Their high degree of specificity to target insects and their lack of toxicity to vertebrates, including humans, as well as their being completely biodegradable has resulted in these 3D-Cry toxins being widely used in agriculture, either as a spray or when expressed in transgenic crops to control insect pests; their wide-spread adoption has resulted in decreased use of field-applied chemical pesticides [2].

To exert their toxic effects, these Cry toxins have to be ingested, where they are then solubilized in the target insect midgut to release the protoxin. The protoxin is then proteolytically cleaved to produce activated toxins, which cross the peritrophic membrane and bind to specific receptors on the brush-border membrane of the larval midgut cells. Following binding, the toxin subunits oligomerize to form pore structures and insert into the membrane. These pores allow ions and water to pass freely into cells, causing osmotic imbalance, finally leading to insect mortality [3]. In this pore-formation model, cadherin-like proteins (CADs) are crucial, not only because they serve as specific receptors for these toxins, but they also facilitate the post-binding-specific proteolytic cleavage step that induces toxin oligomerization and subsequent efficient pore formation [4,5,6]. As Cry toxin expressing crops, such as *Bt*-Maize and *Bt*-cotton, are commercially grown on a wide scale and following reports on the emergence of Cry toxin resistance in target insects, either in the laboratory [7,8,9,10,11,12,13] or in the field [14,15,16,17], there is increasing interest in the interactions between CAD and Cry toxins.

CADs represent a large and highly diverse superfamily that is present in both vertebrates and invertebrates. Their functions are also diverse and include cell recognition, cell signaling, cell communication, morphogenesis, angiogenesis, maintenance of cell structure and possibly even neurotransmission [18]. According to their structural and functional properties, CADs can be grouped into three main categories: classical, desmosomal and atypical cadherins, the latter of which includes flamingo cadherins, FAT-family, T-cadherins and protocadherins [19].

In 1993, a 210-kDa Cry1Ab binding protein was purified from the midgut epithelium of a lepidopteran insect, *Manduca sexta* L (Lepidoptera: Sphingidae), and identified as a CAD [20]. In 1995, this protein was cloned [21] and classified as an atypical cadherin from the protocadherin group [19,22]. Subsequently, CADs have been extensively studied as Cry1A receptors and have been identified in many different lepidopteran insects, such as *Heliothis virescens* (Fabricius) (Lepidoptera*:* Noctuidae) [23,24], *Bombyx mori* L (Lepidoptera: Bombycidae) [25,26,27], *Helicoverpa armigera* (Hbn.) (Lepidoptera: Noctuidae) [28], *Pectinophora gossypiella* (Saunders) (Lepidoptera: Gelechiidae) [25] and *Ostrinia nubilalis* (Hbn.) (Lepidoptera: Crambidae) [29]. Alignment of genes encoding CADs in *O. nubilalis*, *H. armigera* and *B. mori* revealed the following basic structures in common: an extracellular domain that contains a signal peptide and 9–12 cadherin repeats (CRs); a membrane proximal extracellular region (MPR); a transmembrane region (TM); and a small cytoplasmic domain (CD). However, this structure does not appear to be present in the three previously established cadherin categories. The classical CADs are usually located within adherent junctions involved in cell-cell adhesion, whereas lepidopteran CADs are usually located at the base of the microvilli along the length of the midgut and at the apical tip of microvilli in the middle and posterior regional membrane of the midgut [30,31], where the Cry1A toxins are also targeted [30,32]. Although the physiological functions of the *Bt*-related CADs described in lepidopteran insects have not been fully elucidated, their distribution increases the contact of membrane CADs with Cry1A-type toxins in the midgut fluid, thus facilitating the binding and toxicity of these toxins.

Cry1A toxin binding regions in the CADs have been reported in many lepidopteran insects, such as *B. mori* [33], *M. sexta* [34,35], *H. armigera* and *P. gossypiella* [36]. Mutations in genes encoding CAD proteins, which affect the toxin binding region to yield shortened proteins, are known to be tightly linked with resistance to Cry1A toxins [23,25,37]. The toxin binding regions play a key role in the interaction between CADs with Cry toxins, where they may either act as synergists [38,39,40,41] or antagonists [42] by enhancing or reducing, respectively, Cry toxicity against lepidopteran, dipteran or coleopteran insect larvae. Not only the mutations, but also downregulation of a midgut CAD (DsCAD1) in resistant (Cry1Ab-RR) strains of *Diatraea saccharalis* (F.) (Lepidoptera: Crambidae) was shown to be functionally correlated with a decrease in Cry1Ab susceptibility, suggesting a strong association between CADs and the resistance to Cry toxins [43].

Asian corn borer (ACB), *Ostrinia furnacalis* (Guenée) (Lepidoptera: Crambidae), is a key insect pest of maize in China. Yield losses to this insect are estimated to be 10%–20%, but may be more than 30% or may even result in no harvest at all in an outbreak year [44,45]. Field trials demonstrate that Cry1Ac-expressing maize can offer effective control of ACB [46]. Using co-immunoprecipitation (Co-IP), we have previously shown that a CAD acts as a putative Cry1A type binding protein in ACB [47]. We have also previously selected for a Cry1Ac-resistant strain of ACB under laboratory conditions, which exhibits various levels of cross-resistance to Cry1Ab, Cry1Ah and Cry1F [48]. The objective of the present study is to understand the potential role of CAD in the development of resistance to the Cry1Ac toxin in ACB.

## 2. Results

### 2.1. cDNA of the Cadherin-Like Protein in Asian Corn Borer (ACB)

**Figure 1:**
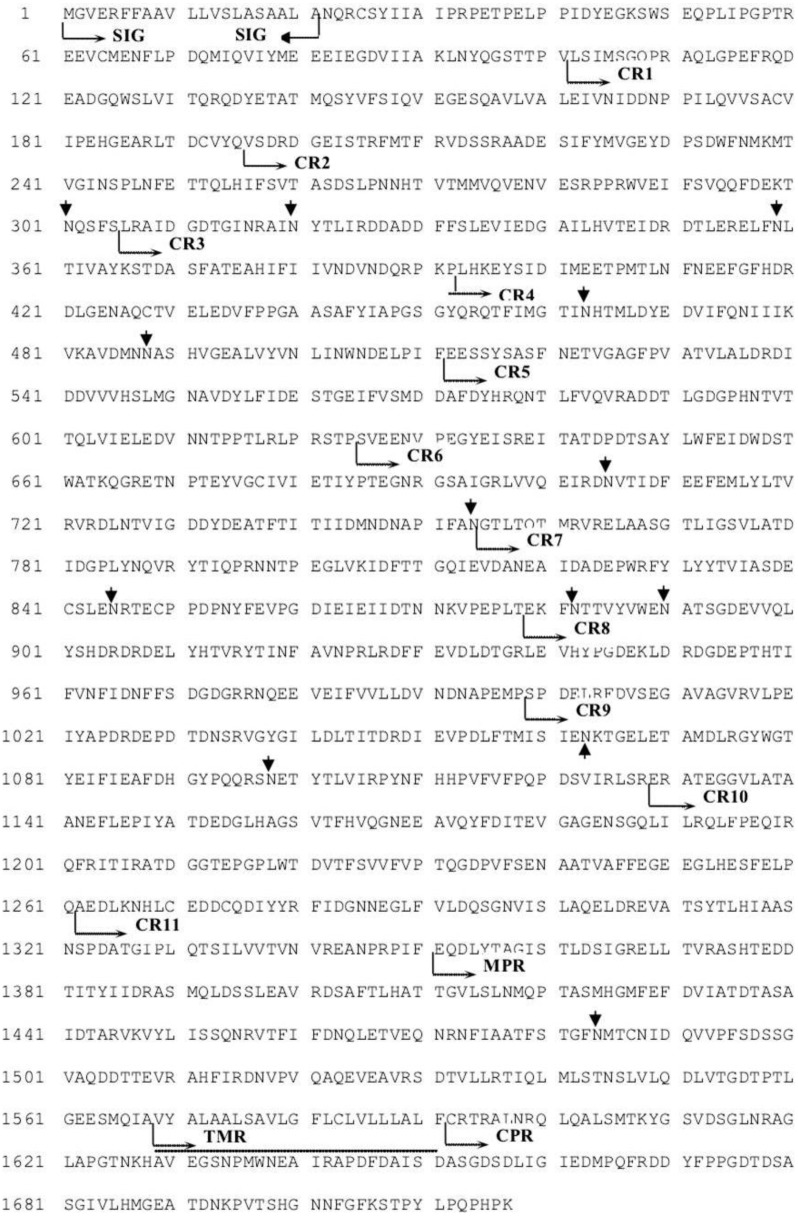
Deduced amino acid sequence of OfCAD (GenBank Accession No. EU022587.1). Protein sequence analysis was carried out using the Swiss Institute for Experimental Cancer Research (ISREC) ProfileScan Server [49]. SIG is the putative signal peptide sequence, CR1-CR11 are cadherin repeats, MPR is the membrane-proximal extracellular region, TMR is the transmembrane spanning region (indicated by a thin underline) and CPR is the small cytoplasmic region. Potential N-glycosylation sites are indicted by arrows.

A cDNA sequence coding for *ofcad* (GenBank Accession No. EU022587.1), a CAD gene from the larval midguts of ACB-*Bt*S, was obtained. The open reading frame (ORF) sequence consisted of 5154 nucleotides and encodes for OfCAD (1717 amino acid residues) with a predicted molecular weight of 191.92 kDa and an isoelectric point of 4.21. The OfCAD was shown to have the common features of lepidopteran CADs, including a signal peptide, which contained 21 amino acids residues, 11 cadherin repeats (CRs), a membrane proximal extracellular region (MPR), a transmembrane region (TMR) and a small cytoplasmic region (CPR) (Figure 1).

### 2.2. Recombinant Expression of OfCAD Internal Peptides and Binding Studies

**Figure 2:**
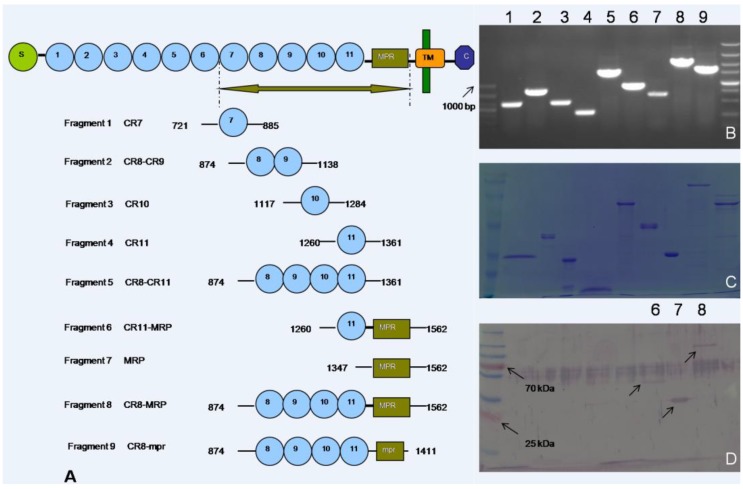
Mapping of the toxin-binding region (TBR) domain of OfCAD. (**A**) Schematic diagram with the terminal amino acid number; (**B**) PCR products of the fragments, which were individually inserted into the pET30a (+) expression vector. Numbers written in bold black are the serial numbers of the nine peptides; (**C**) purified peptides separated by 10% SDS-PAGE visualized by Coomassie Blue staining; peptides designated 1, 3 and 8 were expressed both as soluble proteins and inclusion bodies; Peptide 7 was expressed as a soluble protein, whilst Peptides 2, 4, 5, 6 and 9 were expressed as inclusion bodies; (**D**) ligand blot of purified peptides blotted with Cry1Ac; arrows indicate the presence of peptides binding to Cry1Ac.

Based on the cDNA sequence of cadherin from ACB*-Bt*S, nine cDNA fragments (Figure 2B) were generated by PCR, which were inserted into the pET30a (+) expression vector. Nine recombinant peptides were subsequently expressed and purified (Figure 2C). These peptides matched the predicted molecular weights (Table 1) and could be detected by antibodies against the His-tag, which formed part of the expression construct. Ligand blots revealed the presence of three peptides (Figure 2D), indicating that Cry1Ac had bound to these peptides, designated 6, 7 and 8. Each of these peptides contained the following domains, CR11-MPR, MPR and CR8-MPR, respectively, demonstrating that the minimum binding region mapped to MPR.

**Table 1:** The predicted molecular weights and solubility of the recombinant peptides.

Fragment	1	2	3	4	5	6	7	8	9
No. of amino acid residues	216	315	216	153	538	354	267	739	588
Molecular weight (kDa)	24	36	24	17	60	39	29	82	66
Express in supernatant	+	−	−	−	−	−	+	+	−
Express as inclusion body	+	+	+	+	+	+	−	+	+

### 2.3. MPR Mutants in ACB-Ac200 and Their Binding Ability to Cry1Ac

**Figure 3:**
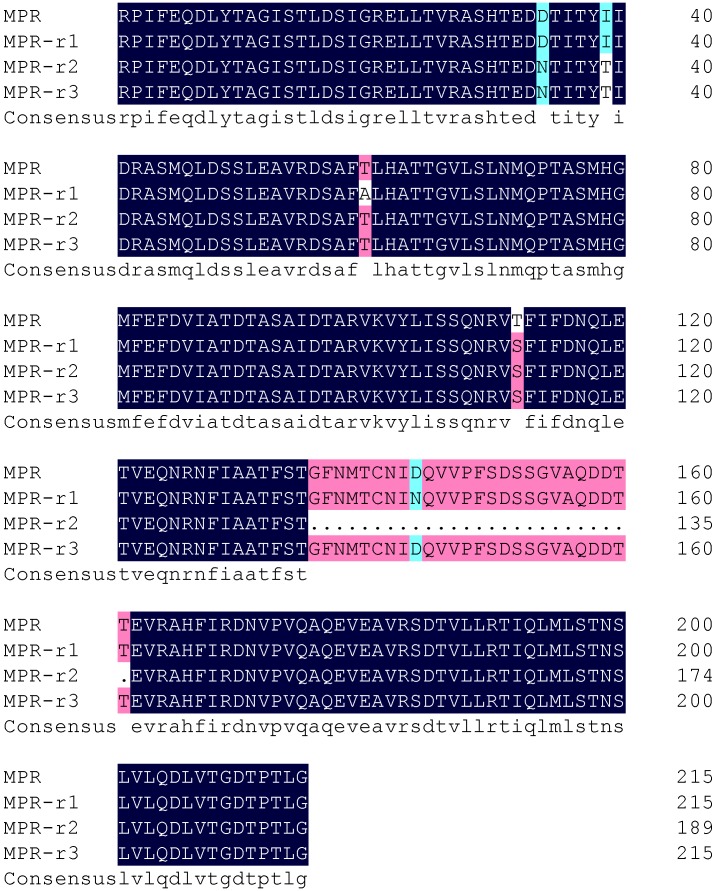
The ACB-Ac200 survivors harboured three mutant alleles in the MPR of OfCAD, namely MPR-r1, MPR-r2 and MPR-r3. The amino acid sequences were aligned with MPR from ACB-*Bt*S using DNAMAN 6.0.

**Figure 4:**
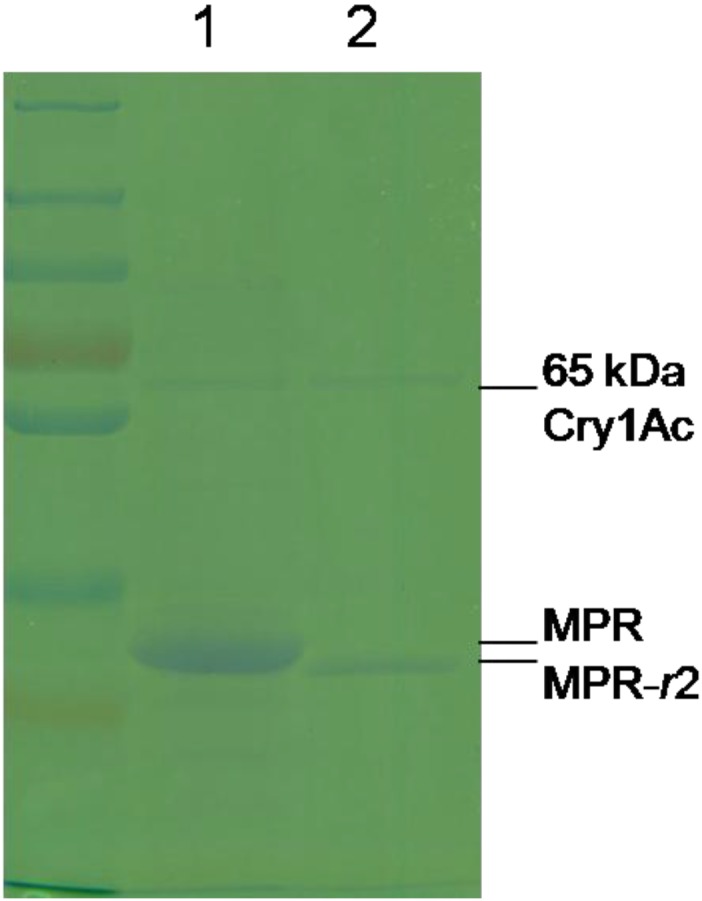
Co-immunoprecipitation assays demonstrating the binding ability of MPR (Lane 1) and MPR-r2 (Lane 2) to Cry1Ac. The fainter band shows that less MPR-*r*2 is able to bind to the same amount of Cry1Ac compared to MPR.

The cDNAs coding for MPR in ACB-Ac200 were obtained by amplification with primers F5/R5 (Table 2). The deduced amino acid sequences of these cDNAs were aligned with the MPR amino acid sequence in ACB-*Bt*S (Figure 3) using DNAMAN, which revealed that they were either amino acid substitutions or omission mutants, *i.e.*, there were two, three and three amino acid substitution mutations in MPR-*r*1, MPR-*r*2 and MPR-*r*3, respectively. The substitution of Thr^1457^→Ser occurred uniformly. In addition, 26 amino acid residues were absent in MPR-*r*2, thus resulting in a lower molecular weight (Figure 4). Differences in the respective binding abilities between MPR-*r*2 and MPR to Cry1Ac were revealed by Co-IP and shown to be weaker in MPR-*r*2 compared to MPR (Figure 4).

**Table 2:** Primers used in this study.

Fragment	Primers	Position	Primer DNA sequence	Product
**For cloning of *ofcad***				
	*ofcad*-F	223	CARSTBATMTWYRTGGAKGARGA	
	*ofcad*-R	4870	TCVACRGYRTGYTTGTTRGTRCC	
	GSP1	792	CGAGTCAGAAGCTGTGACGCT	
	GSP2	536	CAGGCGCTGACCACTTGCAGGAT	
	AUAP	--	CAGGCGCTGACCACTTGCAGGAT	
	*ofcad*-3F	4178	AGACCGTGCGAGCATGCAGCT	
	3 sites adaptor	--	CTGATCTAGAGGTACCGGATCC	
**For generating OfCAD fragments**				
Fragment1	F1	2161	CCGGAATTCCGCGTGAGGGACCTCAACACT	507
	R1	2633	CCGCTCGAGTTACACCGTCGTGTTGAACTTCTCAG	
Fragment2	F2	2623	CCAGAATTCGAGCCGCTCACTGAGAAGTTCAAC	803
	R2	3394	GCCTCGAGTTACGCCAGAACGCCGCCTTCTGT	
Fragment3	F3	3358	CCAGAATTCCCCGACTCCGTCATTCGGCTTTC	507
	R3	3829	CCGCTCGAGTTAGCCGTCAATAAACCTGTAGTAGAT	
Fragment4	F4	3778	CCGGAATTCCCGCAAGCAGAAGACCTTAAAAACCA	308
	R4	4063	CCGCTCGAGTTACGTCGAAATGCCCGCTGTGTA	
Fragment5	F2	2623	CCAGAATTCGAGCCGCTCACTGAGAAGTTCAAC	1473
	R4	4063	CCGCTCGAGTTACGTCGAAATGCCCGCTGTGTA	
Fragment6	F4	3778	CCGGAATTCCCGCAAGCAGAAGACCTTAAAAACCA	921
	R5	4667	CCGCTCGAGTTACTCGCCTAGCGTCGGAGTGT	
Fragment7	F5	4039	CCGGAATTCCGCCCAATTTTCGAGCAGGACCTTTA	660
	R5	4667	CCGCTCGAGTTACTCGCCTAGCGTCGGAGTGT	
Fragment8	F2	2623	CCAGAATTCGAGCCGCTCACTGAGAAGTTCAAC	2076
	R5	4667	CCGCTCGAGTTACTCGCCTAGCGTCGGAGTGT	
Fragment9	F2	2623	CCAGAATTCGAGCCGCTCACTGAGAAGTTCAAC	1623
	R6	4213	CCGCTCGAGTTAGGTGGTCGCATGCAGCGTGAA	
**For qPCR**				
	q*cad*-F	4329	AGCCCGTGTGAAAGTCTAC	255
	q*cad*-R	4565	ACGGCTTCGACCTCTTGTG	
	qβ-F		CCTCCACCTCCCTCGAGAAG	
	qβ-R		GGCAACGGAACCTCTCGTTA	

### 2.4. Transcription Levels of ofcad

**Figure 5:**
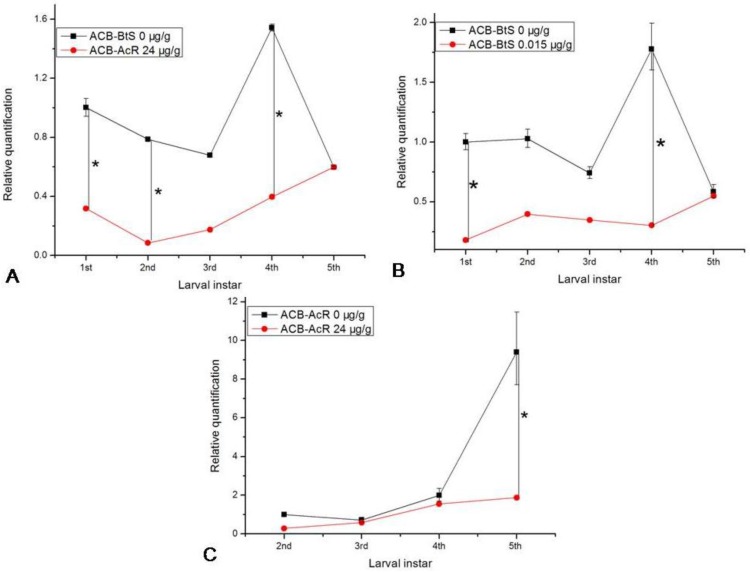
Expression levels of cadherin-like protein gene *ofcad* transcripts during larval development of ACB-*Bt*S and ACB-AcR strains fed on either the control diet or the Cry1Ac toxin containing diet. (**A**) ACB-*Bt*S larvae were reared on control diet and ACB-AcR larvae were reared on Cry1Ac toxin containing diet (24 μg/g); (**B**) ACB-*Bt*S larvae were reared on control and Cry1Ac containing diet (0.015μg/g), respectively; (**C**) ACB-AcR larvae were reared on control diet and Cry1Ac toxin containing diet, respectively.

Expression of the *ofcad* transcripts from the ACB-*Bt*S and ACB-AcR strains during larval development (first to fifth larval instars) was investigated by qPCR (Figure 5). In the ACB-*Bt*S strain, the levels of *ofcad* transcripts were significantly (*F* = 4.23; df = 1,4; *P* = 0.017) greater in larvae fed the control diet compared to when fed the diet containing a sub-lethal dose of Cry1Ac toxin (0.015 μg/g, Cry1Ac/diet; LC_50_ = 0.03 μg/g) (Figure 5B). With the exception of the fourth instar larvae, where transcription levels increased by approximately two-fold, there was an overall decline in *ofcad* transcripts in larvae fed the control diet. This contrasts with larvae fed the sub-lethal dose, where the trend was for a slight increase in transcript level throughout development. Interestingly, there was no significant difference in the number of *ofcad* transcripts in the fifth instar larvae of the ACB-*Bt*S strain, irrespective of diet (Figure 5A,B). These results are in contrast to those for the ACB-AcR strain, where there were no significant differences in *ofcad* transcript levels between those fed control diet and those fed Cry1Ac toxin from the second to fourth instars (Figure 5C). However, by the fifth instar, the *ofcad* transcript levels in the control fed larvae increased approximately five-fold compared to those fed the diet containing Cry1Ac (Figure 5A). Furthermore, the results also showed that transcription levels for *ofcad* were much lower during the first to fourth larval instars when fed the diet containing Cry1Ac, irrespective of the strain of ACB (*i.e.*, ACB-AcR larvae fed Cry1Ac selecting diet and ACB-*Bt*S larvae fed a sub-lethal dose of Cry1Ac toxin) compared to ACB-*Bt*S larvae fed the control diet (Figure 5A,B).

## 3. Discussion

**Figure 6:**
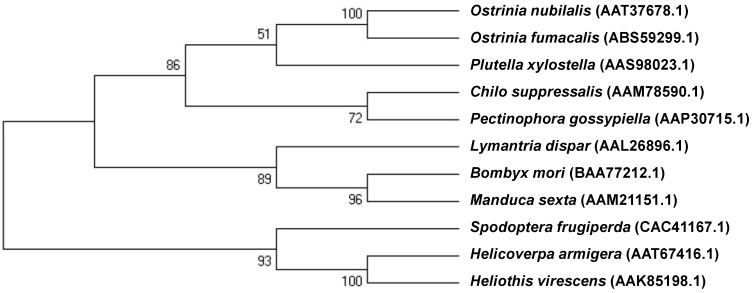
A phylogenetic tree generated by ClustalW alignment of CAD amino acid sequences from insect species using MEGA. The bootstrap values, expressed as percentages of 500 replications, are shown at branch points. GenBank accession numbers are displayed within the tree. Evolutionary analyses were conducted in MEGA5 [50].

A phylogenetic tree generated by ClustalW alignment of CAD amino acid sequences from 11 different lepidopteran insect species showed that the OfCAD identified from ACB exhibited a high degree of similarity to other members of the cadherin super-family in lepidopteran species (Figure 6). Pairwise distance was also analysed to estimate the evolutionary divergence between these sequences [50]. The overall average was 0.434, ranging from 0.013 (*O. furnacalis—O. nubilalis*) to 0.627 (*O. furnacalis—S. frugiperda*).

Previous studies have reported the presence of two Cry1A binding sites in *M. sexta* *Bt*-R1, designated TBR1 (^865^NITIHITDTNN^875^) and TBR2 (^1323^IPLPASILTVTV^1342^), which interact with loop α2 and loop α8 of domain II of the 3D toxin [35]. Similarly, in *O. nubilalis*, these Cry1A binding sites have been shown to be located on CR7 (^861^DIEIEIIDTNN^871^) and CR11 (^1328^IPLQTSILVVTV^1339^), but with several amino acid substitutions [51]. Results from the present study showed that the homolog in *O. furnacalis* was also within the CR7 and CR11 domain, with the same amino acid residues as for *O. nubilalis*. In order to investigate the ability of these two regions to bind the Cry1Ac toxin, both the CR7 and CR11 domains of ACB OfCAD were expressed as recombinant proteins in *E. coli*. However, the results failed to show any binding in ligand blot assays. Similar findings have been reported for both pink bollworm [36] and *M. sexta* [34,52]. In the pink bollworm, the homologs of TBR1 and TBR2 (located in domain CR6 and CR10, respectively) were also expressed in *E. coli*, but likewise, both peptides failed to demonstrate any binding of Cry1Ac under denaturing conditions, although the recombinant CR10 peptide did show some binding in dot binding assays; however, it is not clear whether this is the result of non-specific binding or possible binding to breakdown products.

In *H. virescens*, residues ^1412^GVLSLNMQ^1418^ were previously reported as crucial for binding to Cry1Ac toxin, based on analyses using hydropathic complementarity to the loop 3 region of Cry1Ac [53]. Based on alignment studies, similar homologous peptides were identified in *M. sexta* and *H. armigera* as ^1416^GVLTLNIQ^1423^ and ^1423^GVLSLNFQ^1430^, respectively. Furthermore, in *H. armigera*, the Cry1Ac toxin binding region has been mapped to residues 1,217 to 1,461 [28]. These results are in broad agreement with those of the present study, where the minimum binding region was mapped to MPR and consisted of the following eight amino acid residues, ^1412^GVLSLNMQ^1419^. The importance of these eight amino acid residues in Cry1Ac binding was confirmed by the loss of binding ability following deletion of this epitope in the ninth peptide. This finding corroborates previous studies [53].

Evolution of resistance in target pests to Cry toxins threatens the durability of *Bt* crops. To date, field resistance has been reported for *Bt* corn [54]. One of the most important mechanisms of insect resistance to *Bt* Cry toxins is due to mutation of the receptor in the insect midgut, thus affecting the binding of the *Bt* toxin [11]. Thus, identification of receptors is fundamental, both for understanding the mode of action of *Bt* Cry toxins and the molecular mechanisms of insect resistance to these particular toxins. Cry1A-binding proteins have been widely identified as CADs. Although the relative role of these putative receptor molecules in insects has yet to be conclusively confirmed, Cry1A resistance in a number of lepidopteran insects has been attributed to changes in these cadherin receptors. Such changes may be due to: (1) alternations in expression of CADs, as reported for Cry1Ab resistance in *D. saccharalis* [43] and Cry1Ac resistance in *H. armigera* [28]; (2) mutation of CAD genes, resulting from amino acid substitutions in the TBR of CADs, as reported in *H. virescens* [53]; (3) mutation of the CAD genes resulting from amino acid residue deletions, such as reported for the laboratory selected Cry1Ac resistant strain, GYBT, and the field strain of *H. armigera* [55], as well as in the laboratory selected Cry1Ab resistant strain of *O. nubilalis* [56]; (4) mutation of CAD genes resulting from premature stop codons, such as in *H. virescens* [23]. Further examples also include the laboratory selected Cry1Ac resistant strain of *H. armigera* [37,57], the field evolved Cry1Ac resistant population of *P. gossypiella* [25] and the laboratory selected Cry1Ab resistant strain of *O. nubilalis* [56].

In the present study, screening of cadherin-receptor gene mutants in ACB-AcR focused on the Cry1Ac TBR, which was based on the hypothesis that resistance might result from mutation in this region. Alignment of OfCAD from ACB-AcR and ACB-*Bt*S showed that there were only three amino acid substitutions (D^1379^→N, I^1384^→T and T^1457^→S) in the MPR. However, a mutant with 26-amino acid residue deletions in the MPR (MPR-*r*2) was detected in ACB-Ac200 larvae. Furthermore, the ability of binding with Cry1Ac was significantly reduced in the MPR-*r*2 compared with MPR. These findings suggest that Cry1Ac resistance in ACB is associated with mutations in the TBR of the OfCAD and that the number of amino acid residue deletions influences the level of resistance, as seen for ACB-Ac200 larvae, which had 26 such deletions in the MPR.

Despite the fact that most studies to date have demonstrated that Cry1A resistance in Lepidoptera is linked to mutations in the CAD genes [23,25,37,53,55,56,57,58], a few studies have suggested that resistance in *D. saccharalis* and *H. armigera* to Cry1Ab [43] and Cry1Ac [28], respectively, is due to downregulation of genes encoding CADs. Similarly, results from the present study have shown a significant reduction in transcription levels of *ofcad*, *i.e.*, *ofcad* in the midgut of ACB during the first to fourth larval instars from both the Cry1Ac resistant strain (ACB-AcR) larvae and ACB-*Bt*S larvae exposed to the Cry1Ac toxin. Theoretically, this reduction in expression of OfCAD in ACB-*Bt*S larvae exposed to sub-lethal doses of Cry1Ac toxin may be a stress-induced response as a consequence of an unsuitable food source. Moreover, the reduced expression of OfCAD in ACB-AcR larvae fed on a normal diet was also responsible for a fitness cost in terms of longer development period, reduced pupal masses, *etc.* These results suggest that Cry1Ac resistance in ACB is primarily associated with the downregulation of CADs.

## 4. Experimental Section

### 4.1. Insect Strains and Experimental Treatments

Two strains of Asian corn borer (ACB), *O. furnacalis* (Guenée), were used in this study, *i.e.*, a *Bt*-susceptible strain (ACB-*Bt*S) and a Cry1Ac-resistant strain (ACB-AcR). ACB-*Bt*S larvae were reared on a semi-artificial diet as described by Zhou *et al.* [59]. ACB-AcR larvae were selected and maintained using activated Cry1Ac toxin as described by Han *et al*. [48]. An ACB-Ac200 colony was selected from ACB-AcR by exposing neonates to a diet containing Cry1Ac toxin at a concentration of 200 μg/g for 7 days. All survivors (12 out of 96 larvae exposed to Cry1Ac at a concentration of 200 μg/g) were then transferred to a new agar-free semi-artificial diet without Cry1Ac and were then reared on this diet until the 4th instar.

### 4.2. Cry1Ac Toxin

**Figure 7:**
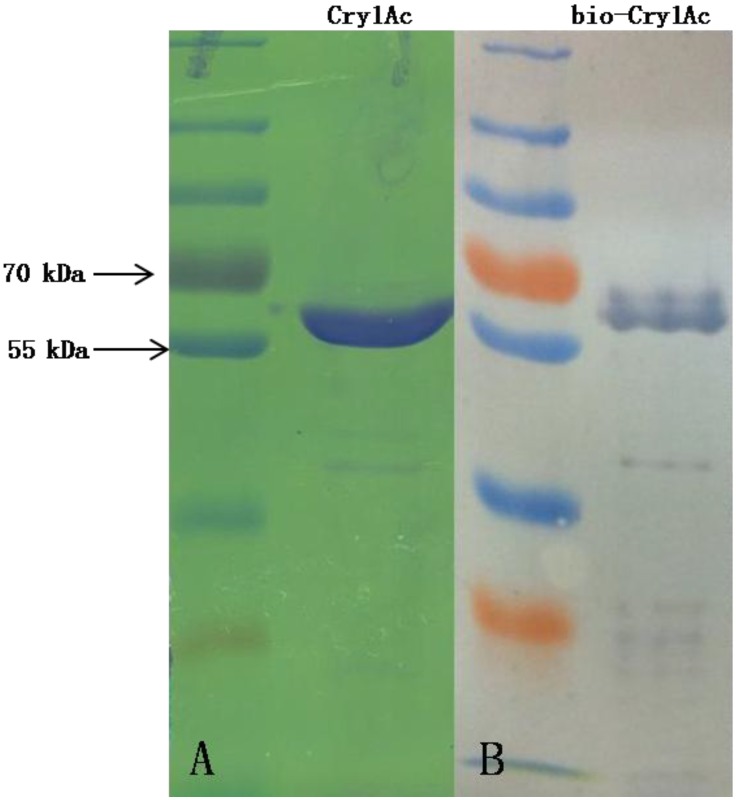
The purity of Cry1Ac (**A**) used in this study was demonstrated by SDS-PAGE, where the separated protein (2 μg Cry1Ac) was stained with Coomassie Blue. The binding activity of the bio-Cry1Ac (**B**) was demonstrated by the transfer of 1 μg bio-Cry1Ac, following SDS-PAGE, onto nitrocellulose membrane with an iBlot™ Gel Transfer Device (Invitrogen, Carlsbad, CA, USA) and developed by the Western Blue^®^ Stabilized Substrate for Alkaline Phosphatase (Promega, Madison, WI, USA).

Activated, salt-free Cry1Ac toxin was purchased from Envirotest-China (Figure 7A) and biotinylated using EZ-Link NHS-Biotin Reagents (Thermo Fisher Scientific, Rockford, IL, USA) following the manufacturer’s instructions (Figure 7B). Excess non-reacted and hydrolysed biotin reagent was removed using Zeba Spin Desalting Columns (Thermo Fisher Scientific, Uppsala, Sweden).

### 4.3. ACB Larval Midgut cDNA Synthesis

Total RNAs were isolated using the RNeasy Mini Kit (Qiagen, Hilden, Germany) from the midguts of larvae from the 1st to the 5th instars. At least 12 larvae were used for each experiment. The first strand cDNA used for PCR was synthesized using the TransScript One-Step gDNA Removal and cDNA Synthesis SuperMix Kit (Transgen, Beijing, China). For the construction of the expression plasmid for OfCAD fragments, total RNA extracted from ACB-*Bt*S served as the template to synthesise the cDNA. For screening the MPR mutants, total RNA isolated from the ACB-Ac200 colony served as the template. For qPCR, the total RNAs extracted from larvae of the 1st to 5th instar from both ACB-*Bt*S (fed on a diet either containing Cry1Ac toxin at 0.015 μg/g (sub-lethal dose) or no toxin) and ACB-AcR (fed on a diet either containing Cry1Ac toxin at 24 μg/g (ACB-AcR selecting dose) or no toxin) served as templates, with the exception of 1st instar ACB-AcR larvae (fed on the diet without Cry1Ac), where RNA was not available.

### 4.4. Cloning of a Full-Length cDNA Coding for Cadherin-Like Proteins in ACB

The full-length cDNA coding for OfCAD was cloned by RACE using the 5’ RACE System for Rapid Amplification of cDNA Ends (Invitrogen, USA) and 3’-Full RACE Core Set (Takara, Dalian, China). Degenerate primers (*ofcad*-F and *ofcad*-R) were used to obtain a middle fragment, and the GSPs (GSP1, GSP2 and *ofcad*-3F) combined with the primers supplied by the kits (AUPU and 3 sites adaptor) were used to amplify both ends of the cDNA. Primers (Table 2) used in this section were designed by Oligo 6 (Primer Analysis Software, version 6.71, Wojciech & Piotr Rychlik) and synthesized by Sangon Biotech Co. Ltd. (Shanghai, China).

In both the 5’ and 3’ RACE reactions, PCR products were subcloned into the pEASY-T1 simple cloning vector (Transgen, Beijing, China), transformed into Trans1-T1 Phage Resistant Chemically Competent Cells (Transgen, Beijing, China) and cultured on LB solid agar plates according to the manufacturer’s instructions. Positive recombinant plasmids were sequenced by Sangon Biotech Co. Ltd. (Shanghai, China). The sequences were analysed by DNAMAN 6, and full-length cDNA sequences were assembled. Protein sequence analysis was carried out using the ISREC Profile Server [49], and the presence of the signal peptide was tested by the SignalP Server [60]. Various physical and chemical parameters were computed using ProtParam [61]. N-Glycosylation sites were predicted using the NetNGlyc Server [62].

### 4.5. Expression and Purification of OfCAD Fragments

Primers used to construct the expression vectors were designed based on the cDNA of *ofcad* (Accession No. EU022587.1) cloned in this study using Oligo 6 (see Table 2). PrimeSTAR^®^ HS (Premix) (Takara, Dalian, China) was used to clone the *ofcad* fragments. PCR products were purified by the EasyPure PCR Purification Kit (Transgen, Beijing, China), digested with *EcoR1* and *Xho1* restriction endonuclease (NEB), inserted into the expression vector pET30a(+) (Novagen, Darmstadt, Germany) using the DNA Ligation Kit Ver.2.1 (Takara, Dalian, China) and transformed into Trans1-T1 Phage Resistant Chemically Competent Cells (Transgen, Beijing, China) following the manufacturer’s instructions. The recombinant plasmids were sequenced and transformed into Trans BL21 (DE3) Chemically Competent Cells (Transgen, Beijing, China). The target peptides were expressed in *E. coli* (ArtMedia Protein Expression; Transgen, Beijing, China) containing Kanamycin selection and affinity purified on a Ni-Agarose column (CWBIO, China; CW0894 was used to purify the soluble proteins; CW0893 was used to purify the proteins expressed as inclusion bodies). The purified peptides were concentrated using Centricon-10 centrifugal filters (Millipore, Ireland), separated by 10% SDS-PAGE gel electrophoresis and detected by staining with Coomassie Brilliant Blue. Recombinant protein was desalted using Zeba Desalt Spin Columns (Thermo Scientific, Uppsala, Sweden) and the protein concentration determined using the Easy Protein Quantitative Kit (Transgen, Beijing, China).

### 4.6. Ligand Blot Binding Assay

Each (1 μg) of 9 purified peptides were separated by 10% SDA-PAGE and transferred onto nitrocellulose membranes using iBlot™ Gel Transfer Device (Invitrogen, Carlsbad, CA, USA). After blocking in TBST buffer (CWBIO, Beijing, China) containing 3% BSA for 1 h, membranes were incubated with 20 nM Cry1Ac for 2 h and probed with rabbit antiserum against Cry1A (1:2000 dilution) in TBST buffer for 1 h. Goat anti-rabbit IgG, AP conjugate (CWBIO, Beijing, China), was used as the secondary antibody (1:5000 dilution) and developed using Western Blue^®^ Stabilized Substrate for Alkaline Phosphatase (Promega, Madison, WI, USA). Membranes were washed between each step (3 times), 5 min in TBST buffer. All steps were conducted at room temperature on an orbital shaker.

### 4.7. Variation of Membrane Proximal Extracellular Region (MPR) Screening in ACB-Ac200 Larvae

F5/R5 (Table 2) served as primers to clone the MPR of the ACB-Ac200 colony; the cDNA used in this section was as described in Section 4.3. PrimeSTAR^®^ HS DNA Polymerase was used here; an extra A was added to the PCR product by incubating with the PCR products at 72 °C for 30 min. The PCR products were then subcloned, transformed and sequenced following the method described in Section 4.4. Bitraditional sequencing was used to verify the cDNAs.

### 4.8. Binding Assay through Co-Immunoprecipitation (Co-IP)

Co-IP was used to detect the difference in the binding ability of Cry1Ac between the recombinant expressed MPR of the ACB-*Bt*S strain and the mutant allele (MPR-r2) screened in Section 4.7. This additional binding assay was carried out, since, in contrast to ligand blots (which are carried out under denaturing conditions, so as to completely expose the epitope), Co-IP is carried out under non-denaturing conditions.

Dynabeads^®^ M-280 Streptavidin (Invitrogen Dynal, Oslo, Norway) was coated with bio-Cry1Ac. The immobilized target protein was then incubated with surplus recombinant MPR or MPR-r2 (previously expressed in *E. coli*), and the captured peptides were dissociated by boiling, following the manufacturer’s instructions. Samples were separated by 10% SDS-PAGE and stained with Coomassie Brilliant Blue.

### 4.9. Quantitative Real-Time PCR (qPCR)

To further compare the transcription levels of *ofcad* in both ACB-*Bt*S and ACB-AcR strains, qPCR was performed with a set of cDNAs (described in Section 4.3), which served as templates. Amplifications were conducted with three technical replicates for each of the two independent biological samples on an ABI7500 thermal cycler (ABI, Abilene, TX, USA), with β-actin as a reference. The SYBR Premix Ex Taq™ kit (Takara, Dalian, China) was used in qPCR; reaction samples (20 μL) consisted of 10 μL of SYBR Premix Ex Taq (Takara, Dalian, China), following the manufacturer’s instructions. Relative transcript abundance was calculated using the 2^−ΔΔ*C*T^ method [63]. Data from gene expression assays were analysed using analysis of variance (ANOVA), and the means were separated by Fisher’s protected LSD test for significance, using the SAS program (SAS Institute Inc., Cary, NC, USA, 1999). The primers used (see Table 2) were validated as described by Livak *et al.* [63].

## 5. Conclusions

The present study identifies a CAD gene *ofcad* from the larval midguts of ACB-BtS. The open reading frame (ORF) sequence of *ofcad* consists of 5154 nucleotides and encodes a cadherin-like protein (OfCAD; 1717 amino acid residues), with a predicted molecular weight of 191.92 kDa and an isoelectric point of 4.21. OfCAD has a high degree of similarity to other members of the cadherin super-family in lepidopteran species. The binding region of the Cry1Ac toxin is located in the membrane proximal extracellular region (MPR) of OfCAD. Cry1Ac resistance in laboratory selected ACB is associated with mutations resulting from amino acid substitutions and/or deletions in this toxin-binding region (TBR). The number of amino acid residue deletions influences the level of resistance, as seen for ACB-Ac200 larvae, which has 26 such deletions in the MPR. In addition, Cry1Ac resistance in ACB is primarily associated with the downregulation of CADs.
